# Privacy and artificial intelligence: challenges for protecting health information in a new era

**DOI:** 10.1186/s12910-021-00687-3

**Published:** 2021-09-15

**Authors:** Blake Murdoch

**Affiliations:** Health Law Institute, Faculty of Law, University of Alberta, Edmonton, AB T6G 2H5 Canada

**Keywords:** Privacy, Artificial intelligence, Bioethics, Health law

## Abstract

**Background:**

Advances in healthcare artificial intelligence (AI) are occurring rapidly and there is a growing discussion about managing its development. Many AI technologies end up owned and controlled by private entities. The nature of the implementation of AI could mean such corporations, clinics and public bodies will have a greater than typical role in obtaining, utilizing and protecting patient health information. This raises privacy issues relating to implementation and data security.

**Main body:**

The first set of concerns includes access, use and control of patient data in private hands. Some recent public–private partnerships for implementing AI have resulted in poor protection of privacy. As such, there have been calls for greater systemic oversight of big data health research. Appropriate safeguards must be in place to maintain privacy and patient agency. Private custodians of data can be impacted by competing goals and should be structurally encouraged to ensure data protection and to deter alternative use thereof. Another set of concerns relates to the external risk of privacy breaches through AI-driven methods. The ability to deidentify or anonymize patient health data may be compromised or even nullified in light of new algorithms that have successfully reidentified such data. This could increase the risk to patient data under private custodianship.

**Conclusions:**

We are currently in a familiar situation in which regulation and oversight risk falling behind the technologies they govern. Regulation should emphasize patient agency and consent, and should encourage increasingly sophisticated methods of data anonymization and protection.

## Background

Advances in healthcare artificial intelligence (AI) are occurring rapidly and will soon have a significant real-world impact. Several new AI technologies are approaching feasibility and a few are close to being integrated into healthcare systems [1, 2]. In radiology, AI is proving to be highly useful for the analysis of diagnostic imagery [3, 4]. For example, researchers at Stanford have produced an algorithm that can interpret chest X-rays for 14 distinct pathologies in just a few seconds [5]. Radiation oncology, organ allocation, robotic surgery and several other healthcare domains also stand to be significantly impacted by AI technologies in the short to medium term [6–10]. In the United States, the Food and Drug Administration (FDA) recently approved one of the first applications of machine learning in clinical care—software to detect diabetic retinopathy from diagnostic imagery [11, 12]. Because of this rapid progress, there is a growing public discussion about the risks and benefits of AI and how to manage its development [13].

Many technological discoveries in the field of AI are made in an academic research environment. Commercial partners can be necessary for the dissemination of the technologies for real world use. As such, these technologies often undergo a commercialization process and end up owned and controlled by private entities. In addition, some AI technologies are developed within biotechnology startups or established private companies [14]. For example, the noted AI for identifying diabetic retinopathy is developed and maintained by startup IDx [12, 13]. Because AI itself can be opaque for purposes of oversight, a high level of engagement with the companies developing and maintaining the technology will often be necessary. The United States Food and Drug Administration, are now certifying the institutions who develop and maintain AI, rather than focusing on the AI which will constantly be changing [15]. The European Commission has proposed legislation containing harmonized rules on artificial intelligence [16], which delineate a privacy and data principle of organizational accountability very similar to that found in the European General Data Protection Regulation [17, 18]. Other jurisdictions like Canada have not completed tailoring regulation specific to AI [19]. AI remains a fairly novel frontier in global healthcare, and one currently without a comprehensive global legal and regulatory framework.

The commercial implementation arrangements noted will necessitate placing patient health information under the control of for-profit corporations. While this is not novel in itself, the structure of the public–private interface used in the implementation of healthcare AI could mean such corporations, as well as owner-operated clinics and certain publicly funded institutions, will have an increased role in obtaining, utilizing and protecting patient health information. Here, I outline and consider privacy concerns with commercial healthcare AI, focusing on both implementation and ongoing data security.

## Main text

### Concerns with access, use and control

AI have several unique characteristics compared with traditional health technologies. Notably, they can be prone to certain types of errors and biases [20–23], and sometimes cannot easily or even feasibly be supervised by human medical professionals. The latter is because of the “black box” problem, whereby learning algorithms’ methods and “reasoning” used for reaching their conclusions can be partially or entirely opaque to human observers [10, 18]. This opacity may also apply to how health and personal information is used and manipulated if appropriate safeguards are not in place. Notably, in response to this problem, many researchers have been developing interpretable forms of AI that will be easier to integrate into medical care [24]. Because of the unique features of AI, the regulatory systems used for approval and ongoing oversight will also need to be unique.

A significant portion of existing technology relating to machine learning and neural networks rests in the hands of large tech corporations. Google, Microsoft, IBM, Apple and other companies are all “preparing, in their own ways, bids on the future of health and on various aspects of the global healthcare industry [25].” Information sharing agreements can be used to grant these private institutions access to patient health information. Also, we know that some recent public–private partnerships for implementing machine learning have resulted in poor protection of privacy. For example, DeepMind, owned by Alphabet Inc. (hereinafter referred to as Google), partnered with the Royal Free London NHS Foundation Trust in 2016 to use machine learning to assist in the management of acute kidney injury [22]. Critics noted that patients were not afforded agency over the use of their information, nor were privacy impacts adequately discussed [22]. A senior advisor with England’s Department of Health said the patient info was obtained on an “inappropriate legal basis” [26]. Further controversy arose after Google subsequently took direct control over DeepMind’s app, effectively transferring control over stored patient data from the United Kingdom to the United States [27]. The ability to essentially “annex” mass quantities of private patient data to another jurisdiction is a new reality of big data and one at more risk of occurring when implementing commercial healthcare AI. The concentration of technological innovation and knowledge in big tech companies creates a power imbalance where public institutions can become more dependent and less an equal and willing partner in health tech implementation.

While some of these violations of patient privacy may have occurred in spite of existing privacy laws, regulations, and policies, it is clear from the DeepMind example that appropriate safeguards must be in place to maintain privacy and patient agency in the context of these public–private partnerships. Beyond the possibility for general abuses of power, AI pose a novel challenge because the algorithms often require access to large quantities of patient data, and may use the data in different ways over time [28]. The location and ownership of servers and computers that store and access patient health information for healthcare AI to use are important in these scenarios. Regulation should require that patient data remain in the jurisdiction from which it is obtained, with few exceptions.

Strong privacy protection is realizable when institutions are structurally encouraged to cooperate to ensure data protection by their very designs [29]. Commercial implementations of healthcare AI can be manageable for the purposes of protecting privacy, but it introduces competing goals. As we have seen, corporations may not be sufficiently encouraged to always maintain privacy protection if they can monetize the data or otherwise gain from them, and if the legal penalties are not high enough to offset this behaviour. Because of these and other concerns, there have been calls for greater systemic oversight of big data health research and technology [30].

Given we have already seen such examples of corporate abuse of patient health information, it is unsurprising that issues of public trust can arise. For example, a 2018 survey of four thousand American adults found that only 11% were willing to share health data with tech companies, versus 72% with physicians [31]. Moreover, only 31% were “somewhat confident” or “confident” in tech companies’ data security [28]. In some jurisdictions like the United States, this has not stopped hospitals from sharing patient data that is not fully anonymized with companies like Microsoft and IBM [32]. A public lack of trust might heighten public scrutiny of or even litigation against commercial implementations of healthcare AI.

### The problem of reidentification

Another concern with big data use of commercial AI relates to the external risk of privacy breaches from highly sophisticated algorithmic systems themselves. Healthcare data breaches haven risen in many jurisdictions around the world, including the United States [33, 34], Canada [35–37], and Europe [38]. And while they may not be widely used by criminal hackers at this time, AI and other algorithms are contributing to a growing inability to protect health information [39, 40]. A number of recent studies have highlighted how emerging computational strategies can be used to identify individuals in health data repositories managed by public or private institutions [41]. And this is true even if the information has been anonymized and scrubbed of all identifiers [42]. A study by Na et al., for example, found that an algorithm could be used to re-identify 85.6% of adults and 69.8% of children in a physical activity cohort study, “despite data aggregation and removal of protected health information [43].” A 2018 study concluded that data collected by ancestry companies could be used to identify approximately 60% of Americans of European ancestry and that, in the near future, the percentage is likely to increase substantially [44]. Furthermore, a 2019 study successfully used a “linkage attack framework”—that is, an algorithm aimed at re-identifying anonymous health information—that can link online health data to real world people, demonstrating “the vulnerability of existing online health data [45].” And these are just a few examples of the developing approaches that have raised questions about the security of health information framed as being confidential. Indeed, it has been suggested that today’s “techniques of re-identification effectively nullify scrubbing and compromise privacy [46].”

This reality potentially increases the privacy risks of allowing private AI companies to control patient health information, even in circumstances where “anonymization” occurs. It also raises questions of liability, insurability and other practical issues that differ from instances where state institutions directly control patient data. Considering the variable and complex nature of the legal risk private AI developers and maintainers could take on when dealing with high quantities of patient data, carefully constructed contracts will need to be made delineating the rights and obligations of the parties involved, and liability for the various potential negative outcomes.

One way that developers of AI systems can potentially obviate continuing privacy concerns is through the use of generative data. Generative models develop the ability to generate realistic but synthetic patient data with no connection to real individuals [47, 48]. This can enable machine learning without the long term use of real patient data, though it may initially be needed to create the generative model.

## Conclusions

It is an exciting period in the development and implementation of healthcare AI, and patients whose data are used by these AI should benefit significantly, if not greatly, from the health improvements these technologies generate. Nonetheless, the implementation of commercial healthcare AI faces serious privacy challenges. Given personal medical information is among the most private and legally protected forms of data, there are significant concerns about how access, control and use by for-profit parties might change over time with a self-improving AI. An emphasis on patient agency and consent in the development of regulation in this space would reflect the key legal and ethical values of liberal democracies. For example, requirements for technologically-facilitated recurrent informed consent for new uses of data, where possible, would help to respect the privacy and agency of patients. Also, the right to withdraw data could be clearly communicated and especially made easy to exercise; where feasible, generative data could be used to fill the data gaps created by these agency-driven withdrawals and to avoid de-operationalizing AI systems. Regarding the reidentification issue, there will be a need for new and improved forms of data protection and anonymization. This will require innovation, and there will also be a regulatory component to ensuring that private custodians of data are using cutting edge and safe methods of protecting patient privacy.

We are currently in a situation in which regulation and oversight risk falling behind the technologies they govern. Given we are now dealing with technologies that can improve themselves at a rapid pace, we risk falling very behind, very quickly.

## Data Availability

Not applicable.

## Abbreviation

AI: Artificial intelligence
